# A Conserved Potential Development Framework Applies to Shoots of Legume Species with Contrasting Morphogenetic Strategies

**DOI:** 10.3389/fpls.2017.00405

**Published:** 2017-03-27

**Authors:** Lucas Faverjon, Abraham J. Escobar-Gutiérrez, Isabelle Litrico, Gaëtan Louarn

**Affiliations:** INRA, UR4, URP3F, BP6Lusignan, France

**Keywords:** forage legumes, morphogenesis, branching, architecture, leaf area, growth habit, competitive ability

## Abstract

A great variety of legume species are used for forage production and grown in multi-species grasslands. Despite their close phylogenetic relationship, they display a broad range of morphologies that markedly affect their competitive abilities and persistence in mixtures. Little is yet known about the component traits that control the deployment of plant architecture in most of these species. During the present study, we compared the patterns of shoot organogenesis and shoot organ growth in contrasting forage species belonging to the four morphogenetic groups previously identified in herbaceous legumes (i.e., stolon-formers, rhizome-formers, crown-formers tolerant to defoliation and crown-formers intolerant to defoliation). To achieve this, three greenhouse experiments were carried out using plant species from each group (namely alfalfa, birdsfoot trefoil, sainfoin, kura clover, red clover, and white clover) which were grown at low density under non-limiting water and soil nutrient availability. The potential morphogenesis of shoots characterized under these conditions showed that all the species shared a number of common morphogenetic features. All complied with a generalized classification of shoot axes into three types (main axis, primary and secondary axes). A common quantitative framework for vegetative growth and development involved: (i) the regular development of all shoot axes in thermal time and a deterministic branching pattern in the absence of stress; (ii) a temporal coordination of organ growth at the phytomer level that was highly conserved irrespective of phytomer position, and (iii) an identical allometry determining the surface area of all the leaves. The species differed in their architecture as a consequence of the values taken by component traits of morphogenesis. Assessing the relationships between the traits studied showed that these species were distinct from each other along two main PCA axes which explained 68% of total variance: the first axis captured a trade-off between maximum leaf size and the ability to produce numerous phytomers, while the second distinguished morphogenetic strategies reliant on either petiole or internode expansion to achieve space colonization. The consequences of this quantitative framework are discussed, along with its possible applications regarding plant phenotyping and modeling.

## Introduction

Numerous forage legumes contribute to temperate grasslands and help to supply high-quality protein-rich feed for ruminants, while reducing the need for nitrogen fertilizers (Suter et al., 2015; Vertès et al., 2015), preserving water quality (Owens et al., 1994; Russelle et al., 2001) and mitigating greenhouse gas emissions (Jensen et al., 2012). Most of these legume species are grown in a mixture with perennial grasses in order to take advantage of the ecological and nutritional complementarities of the two functional groups (Nyfeler et al., 2011; Gaba et al., 2015). However, a long-acknowledged problem of multi-species grasslands is the lack of persistence of the legume component over time and a less predictable forage quality when compared with pure grasses or annual forages (Beuselinck et al., 1994; Schwinning and Parsons, 1996). Competition for resources and crop management have been shown to be of considerable importance to regulating the proportion of legumes in grassland communities (Sheaffer, 1989; Beuselinck et al., 1994), but little is known about the mechanisms by which a legume species prevails within a particular community or environment. To date, the search for combinations of traits predictive of legume performance in a mixture has been limited to a few widely-grown mixtures (e.g., white clover-perennial ryegrass or alfalfa-tall fescue: Davies, 2001; Annicchiarico et al., 2015; Maamouri et al., 2015), and only a few studies have sought to explore the role of the diversity of plant forms using multi-trait approaches (e.g., Fort et al., 2015 on root traits; Kraft et al., 2015).

**Figure 1:**
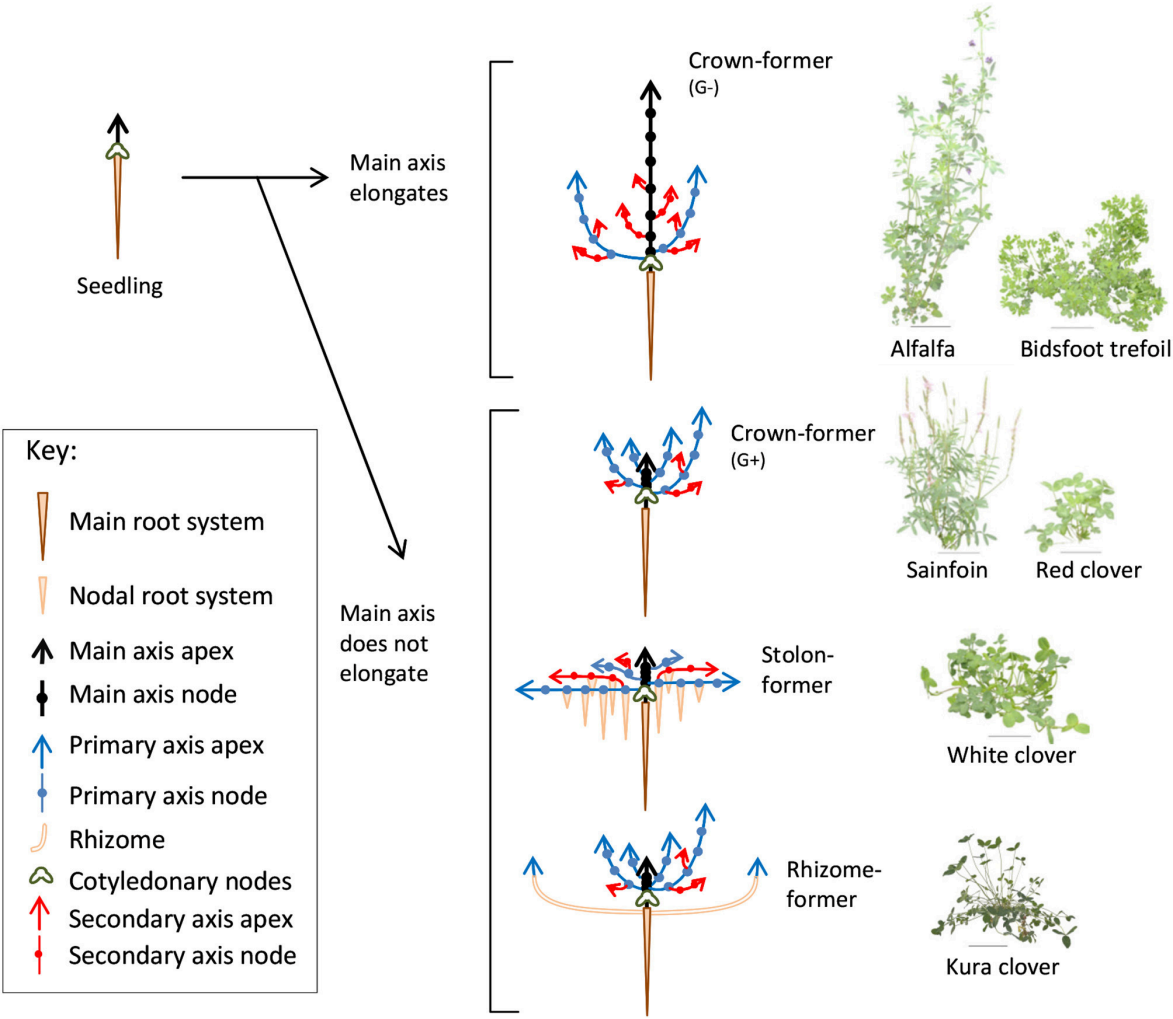
**Diagrammatic representation of the vegetative development of forage legumes from different morphogenetic groups (left) and images of the corresponding species tested (right)**.

The legume family presents spectacular morphological and life-history trait diversity (Lewis et al., 2005; LPWG, 2013). Considering the temperate herbaceous genus only, a remarkable range of plant structures and pedoclimatic adaptations has been reported (Forde et al., 1989; Scott et al., 1989). The morphogenesis of shoots determines plant architecture and light capture (Valladares and Niinemets, 2007), which are critical to inferring the outcome of competition for light (Caldwell, 1987). It also determines the position of shoot meristems and contributes to how different species tolerate grazing and mowing (Briske, 1996; Smith et al., 2000). Differences in leaf surface area, plant height and other architectural features that affect the spatial distribution of leaves (e.g., phenology, branching patterns, dimensions of spacing organs, etc.) have been shown to be drivers of competitive success in grass-legume mixtures (Louarn et al., 2012; Barillot et al., 2014). However, little is known about the elementary traits that promote leaf area expansion and height increments in most herbaceous species, especially regarding the temporal aspects of morphogenesis. Unlike grasses, for which a regular developmental scheme was identified and mobilized a long time ago to compare species and genotypes (Simon and Lemaire, 1987; Lafarge and Durand, 2013), no obvious pattern has emerged from comparisons of shoots from crown-, stolon- and rhizome-forming legumes used for forage production (Forde et al., 1989; Thomas, 2003; Figure 1).

As parts of a modular organism, the common feature of all plant shoots is that they are built from elementary subunits called phytomers (White, 1979). Shoot morphogenesis thus arises from the initiation of new phytomers by shoot meristems, from the expansive growth of the individual organs produced and from the differentiation of support tissues. Like many other dicotyledonous plants (Seleznyova et al., 2002; Lebon et al., 2006), forage legumes are characterized by shoots with complex and highly branched structures (e.g., white clover: Thomas, 1987; Gautier et al., 2000; Thomas et al., 2014; red clover: Taylor and Quesenberry, 1996). Considerable variability of shoot development is usually observed in dense stands (Gosse et al., 1988; Van Minnebruggen et al., 2015). However, architectural and developmental analyses have proved to be powerful tools to classify shoot types and quantify branching and potential shoot development in different species and varieties, based on stochastic (Costes and Guédon, 2002; Louarn et al., 2007) and deterministic (Turc and Lecoeur, 1997) approaches. Although, using different methods, regular organogenetic patterns have been found in alfalfa (Baldissera et al., 2014), red clover (Van Minnebruggen et al., 2014), and Medicago truncatula (Moreau et al., 2006), suggesting that a generalized description could be envisioned for these species.

Similarly, the complexity of plant growth, distributed between individual organs that all vary in size and shape, has been shown to be driven by a number of common determinants. For instance, the temporal sequences of organ growth generally appear to be coordinated at a supra-organ level. In grasses, changes to the phases of leaf growth are triggered by emergence of the previous leaves (Skinner and Nelson, 1994; Fournier et al., 2005; Louarn et al., 2010). In many other species, stable thermal time calendars of development have been reported at the plant and phytomer levels in the absence of stress (Granier et al., 2002; Lecoeur, 2010; Demotes-Mainard et al., 2013). Furthermore, the ultimate sizes attained by organs are highly heritable traits (Annicchiarico et al., 1999), which present relatively conserved ontogenic patterns along the stem under controlled conditions (Ross, 1981). Overall, organogenetic and expansive growth characteristics define and—by their ongoing interactions constrain—the shoot morphogenesis of any particular plant genotype. Trade-offs very often occur (e.g., in grasses between leaf growth and tillering; Nelson, 2000; Barre et al., 2015), which therefore makes it worthwhile analysing these two aspects of morphogenesis together.

In order to facilitate the future characterization of shoot morphogenesis in different legume species and cultivars, this paper was designed to: (i) analyse the elementary traits controlling plant leaf area and height in a selection of six herbaceous species, contrasting in terms of their growth habits and architectures, (ii) challenge the existence of a common framework for vegetative development under non-limiting growing conditions, and (iii) assess the relationships between the traits studied and the concomitant occurrence of trait values which might indicate ontogenic trade-offs in the morphogenesis of shoots.

## Materials and methods

### Plant materials and growing conditions

Three experiments were carried out in a greenhouse at INRA Lusignan, France (46°26′ N, 0°07′ E). The two first took place from February to April in 2014 and 2015, whereas the third experiment was carried out from November 2016 to February 2017. Plants of alfalfa (*Medicago sativa* L. cv. Timbale; hereinafter referred to as A), white clover (*Trifolium repens* L. cv. Giga; WC), red clover (*Trifolium pratense* L. cv. Formica; RC), and sainfoin (*Onobrychis viciifolia* Scop. cv. Canto; SF) were studied during the two first experiments. In addition, plants of birdsfoot trefoil (*Lotus corniculatus* L. cv. Leo; BT) and kura clover (*Trifolium ambiguum* cv. Sevanskij; KC) were grown in the second and third years. Overall, the six species selected covered a wide range of shoot growth habits and the four morphogenetic groups previously reported among perennial herbaceous legumes from temperate areas (Forde et al., 1989; Thomas, 2003; Figure 1) and adapted to contrasting ecological niches (Scott et al., 1989).

In each experiment, seeds from each species were germinated for 48 h in Petri dishes at 25°C in the dark. In addition, clones propagated from rhizome cuttings were selected on 2-years-old plants and used for TK in the third experiment. The seedlings were planted 0.3 m × 1 m apart in custom-built 10 L boxes containing sterile potting mix, sand and brown soil (1:1:1, v/v/v). For each species, the plants were grown in isolation (3.3 plant.m^−2^) until the end of the vegetative stage, according to a randomized block design with four (Exp. 1 and 2) to five (Exp. 3) replicates by species. Irrigation and fertilization were provided throughout the experiments with a drip system 5 cm distant from the plants that delivered 300 mL.d^−1^ of complete nutrient solution (Gastal and Saugier, 1986). The nitrogen concentration of the solution (8 mM) was non-limiting for growth and prevented the nodulation of roots in all the legume species. The greenhouse was heated and a photoperiod of 16 h was maintained by means of 400-Watt HQI lamps (Supplementary Table S1).

### Plant measurements

To analyse the temporal development of photosynthetic surfaces and their spatial distributions in each species, the organogenesis and expansion of shoot organs were measured for all the plants for a period of about 70 days. The terminology used to refer to the different shoot axes was based on the architectural descriptions of alfalfa and white clover (Moulia et al., 2000; Baldissera et al., 2014) and is summarized in Supplementary Figure S1A.

The numbers of axes and numbers of phytomers on the different axes were counted weekly. A decimal scale was used to account for phytomers with unfolded leaves (Maître et al., 1985). Furthermore, several groups of three consecutive phytomers were selected for daily growth measurements on both primary and secondary axes. Ranks 5, 6, 7 and 11, 12, 13 were followed on the main axis. Ranks 3, 4, 5 were characterized on primary axes and branches. The length of each organ (i.e., leaflet, petiole, internode in all species; stipule in birdsfoot trefoil; Supplementary Figure S1B) was measured every day with a ruler until no further growth was noted over 4 consecutive days.

At the end of each experiment, the final length and width of each mature organ along the primary axes was measured. In experiments 1 and 2, a sub-sample of phytomers was used in each species to determine the leaf area of individual organs. Leaves and stipules of various sizes and positions were scanned (Konica Minolta C352/C300, Konica Minolta Sensing, Osaka, Japan) and their area was measured by image analysis (ImageJ software, http://rsbweb.nih.gov/ij/). The height of plants, the total plant leaf area and the total dry weight of plants were also measured.

### Meteorological measurements and thermal time calculations

Relative humidity was measured using a capacitive hygrometer (HMP35A Vaisala, Oy, Helsinki, Finland) and air temperature with copper-constantan thermocouples placed in a ventilated radiation shield at the center of the greenhouse. Photosynthetic photon flux density (PPFD) was also measured by means of PPFD sensors placed above each experimental bloc within the greenhouse. All data were stored in a datalogger (CR10X, Campbell Scientific Ltd.), with measurements taken every 30 s and an average calculated over 15 min. The data are summarized for each experiment in Supplementary Table S1.

Thermal time (*TT*) was calculated as the integral of a non-linear beta function of temperature (*T*) as proposed by Zaka et al. (2017):

(1) $$\begin{array}{l} f\left(T\right)={\left(\frac{T-T_{\text{min}}}{T_{ref}-T_{\text{min}}}\right)}^{q}.\left(\frac{T_{\text{max}}-T}{T_{\text{max}}-T_{ref}}\right) \end{array}$$

(2) $$\begin{array}{l} TT=\overset{t}{\underset{t0}{\int }}\text{max}\left[0,\left(T_{ref}-T_{base}\right).f\left(T\right)\right].dt \end{array}$$

The equation has three parameters: the minimum (T_min_) and maximum (T_max_) temperatures at which development occurs and *q*, a shape parameter. In addition, T_ref_ accounts for a fixed reference temperature (20°C) and T_base_ (5°C) for a common base temperature used to scale time units from equivalent days at the reference temperature into degree days (°Cd). The parameters used for the different species were derived from the literature and are presented in Supplementary Table S2.

### Data analysis

All calculations and statistical tests were performed using R software (version 3.1.2; R Development Core Team, 2014). Rates of leaf appearance were calculated for each axis with three or more unfolded leaves by linear regression between thermal time and the number of visible phytomers. Phyllochrons were calculated as the reciprocal of the leaf appearance rate.

The temporal growth of plant organs was analyzed using a three-parameter logistic function (Equation 3) fitted to the time series of organ growth measurements:

(3) $$\begin{array}{l} L\left(t\right)/L_{\text{max}}=\frac{1}{1+e^{-s.\left(t-t50\right)}} \end{array}$$

where the *s* and *t50* parameters represent the steepness and time delay at mid-organ expansion and *L*_max_ the final organ length. By convention, all the organs within a phytomer were analyzed with respect to the leaf appearance (i.e., *t50* of leaflets = 0) and time was expressed in phyllochrons to aggregate growth series from axes with different developmental rates. The duration of organ expansion between 5 and 95% of its final dimension (*d*_95_) was derived for each organ from the *s* parameter as follow:

(4) $$\begin{array}{l} d_{95}=-2.\text{ln}\left(0.05/0.95\right)/s \end{array}$$

For each phytomer, branching probability was calculated at a given date as the ratio between the number of branches with an outburst at this position and the total number of plants in the treatment. The relationship between branching probability and phytomer position was characterized on the main and primary axes using a logistic function similar to Equation 3.

Significant differences between the means of plant traits were tested by performing analyses of variance (“aov” procedure). Analyses of covariance (ANCOVA, *lm* procedure) were used to test simultaneously for the effects of continuous and categorical variables and to compare the slopes and intercepts of linear relationships. Principal component analysis (PCA) was performed to assess the relationships between shoot morphogenetic parameters using the *ade4* package (13 parameters, 50 individuals).

## Results

### Organogenesis on the main axis

**Figure 2:**
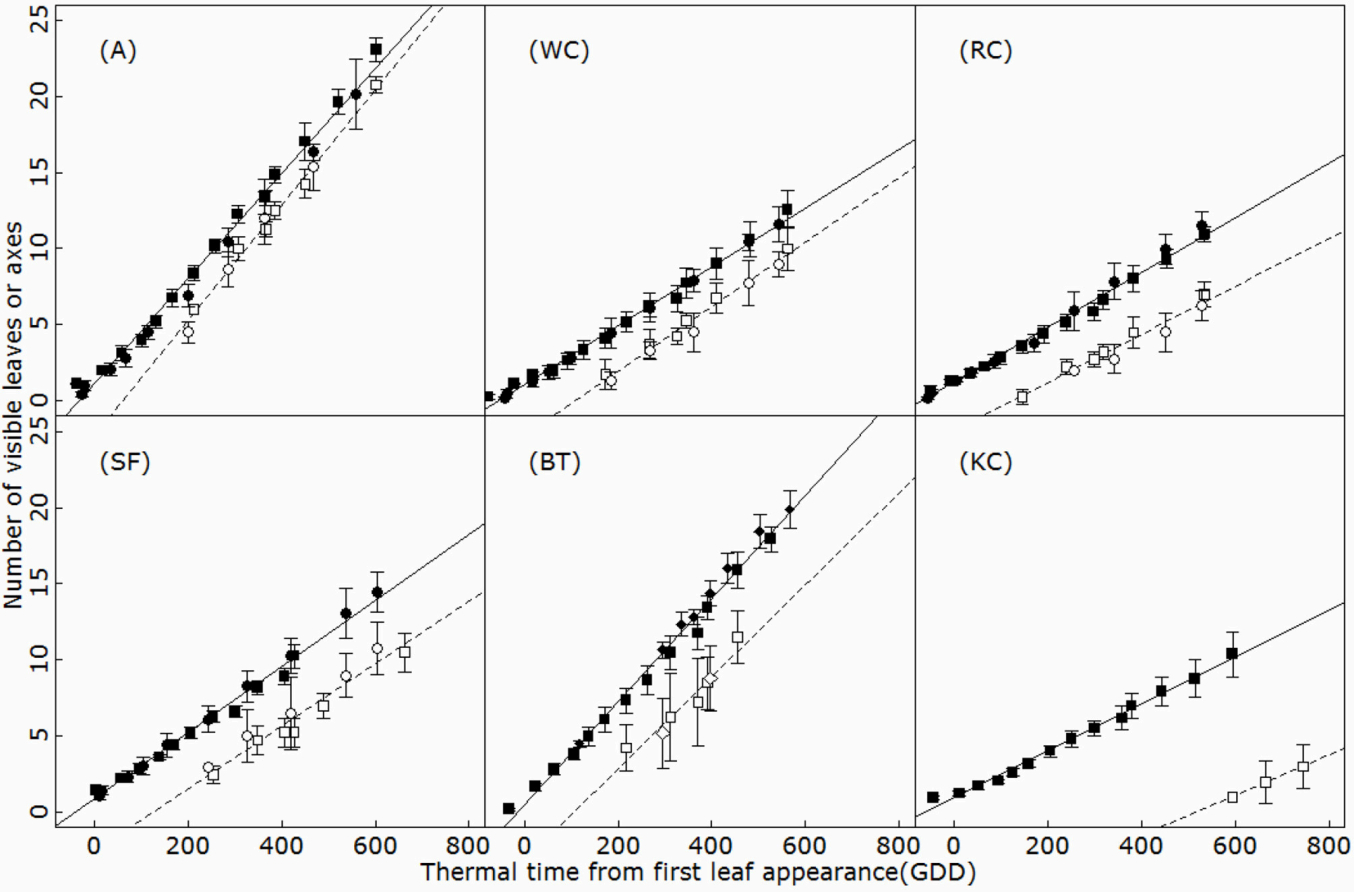
**Timing of leaf (filled symbols) and branch (open symbols) appearance on the main axis of alfalfa (A), white clover (WC), red clover (RC), sainfoin (SF), birdsfoot trefoil (BT), and kura clover (KC)**. Circles: first experiment (2014); squares: second experiment (2015); diamonds: third experiment (2016). GDD: growing °C days. Vertical bars indicate standard deviation (4 to 5 replicates).

New phytomers appeared on the main axis at a constant rate during the vegetative phase, resulting in a linear relationship between the total number of phytomers on an axis and thermal time in all the species studied (*R*^2^ > 0.95; Figure 2). The leaf appearance rate differed markedly between species (ANCOVA, *p* < 0.001) and was conserved between experimental years. Phyllochrons ranged from 32.6°C day in birdsfoot trefoil to 64.2°C day in Kura clover, with a stable pecking order (BT-A<SA<WC<RC<KC).

### Branching

**Figure 3:**
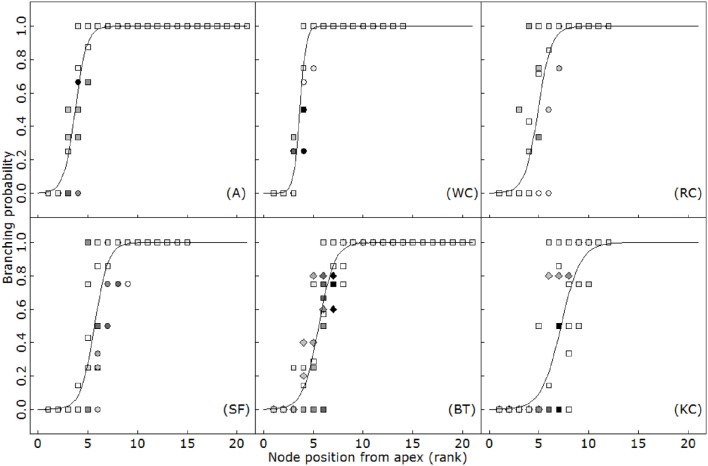
**Branching probability with respect to phytomer position expressed from the apex for alfalfa (A), white clover (WC), red clover (RC), sainfoin (SF), birdsfoot trefoil (BT) and kura clover (KC)**. Circles: first experiment (2014); squares: second experiment (2015); diamonds: third experiment (2016). Different symbols indicate different sampling dates. Logistic curves were fitted using Equation 3.

The timing of budburst of axillary branches on the main axis appeared to be linearly related to thermal time (*R*^2^ > 0.79; Figure 2). A short lag period was observed systematically between the appearance of a phytomer and axillary budburst, so that phytomer appearance and the burst of the corresponding axillary bud was always separated by approximately two (A) to 8 (KC) phyllochrons, depending on the species. This delay of sylleptic branching remained constant over the period of observation in all the species, at all the positions (ANCOVA, *p* > 0.77). Accordingly, branching rates strongly differed between species and followed an inverse phyllochron order (BT-A>SA-WC>RC>KC). Furthermore, sylleptic branching appeared to be systematic on the main axis and primary axes (i.e., the branching probability ultimately reached 1 for all the phytomers after a certain delay; Figure 3), making it a deterministic process in isolated plants subjected to weak competition for light.

### Organogenesis on the primary and secondary axes

**Figure 4:**
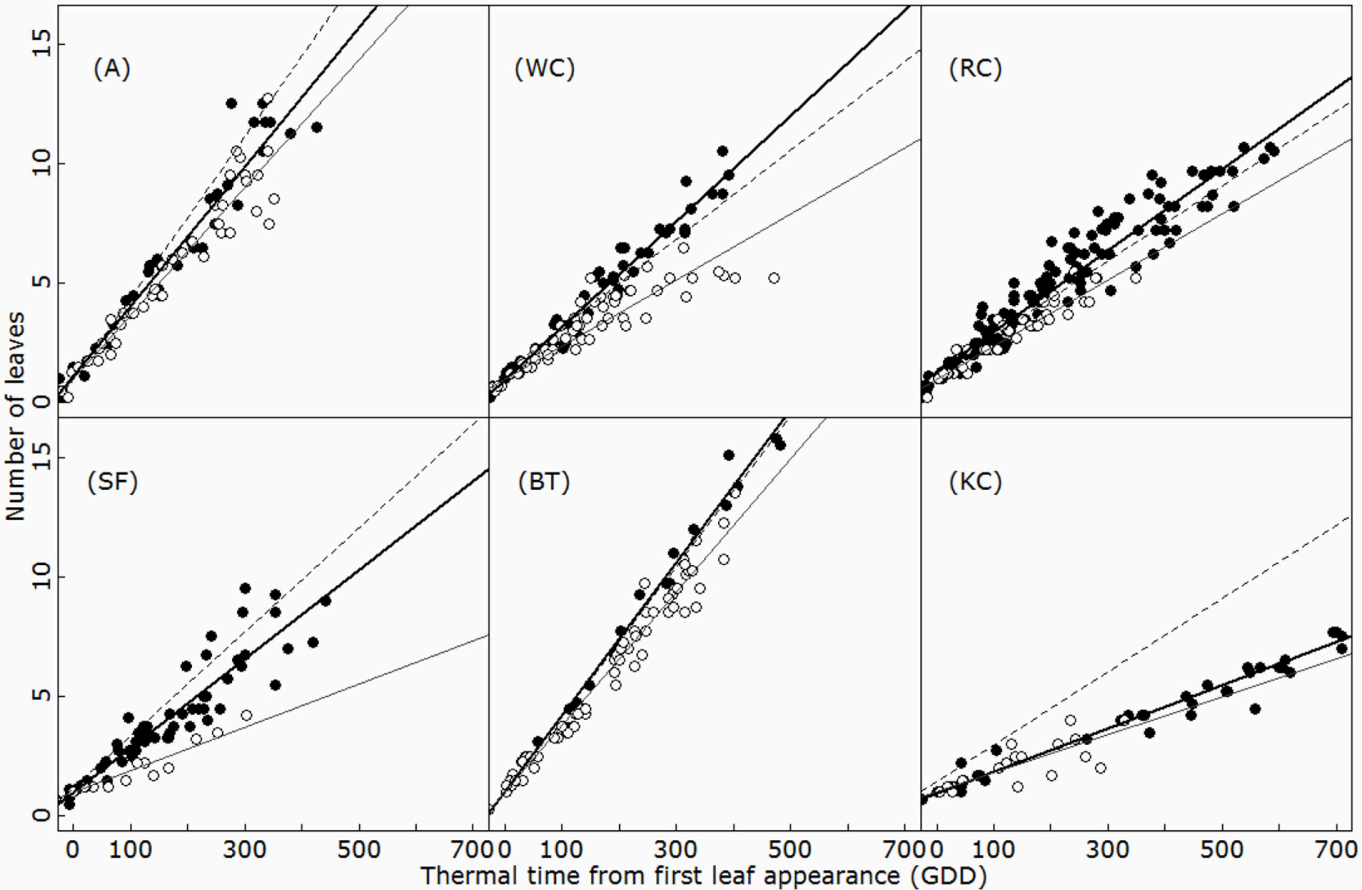
**Leaf appearance on the primary (filled circles) and secondary (open circles) axes for alfalfa (A), white clover (WC), red clover (RC), sainfoin (SA), birdsfoot trefoil (BT), and kura clover (KC)**. Dashed lines indicate the leaf appearance rate on the main axis (Figure 3). Bold and black lines indicate the linear regressions for the primary and secondary axes, respectively. Only axes with at least 3 unfolded leaves were plotted. GDD: growing °C days.

The development of primary (emerging from the collar zone) and secondary axes is further presented in Figure 4. As for the main axis, primary and secondary axes produced new phytomers at a constant rate in thermal time. Leaf appearance rates on the primary axes were similar to the main axis in A and BF, but differed in the other species (ANCOVA, *p* < 0.01). In white and red clovers, the primary axes developed more rapidly than the main axis, whereas the reverse was observed in sainfoin and Kura clover. Comparatively, the secondary axes displayed a much slower rate of development than primary axes (i.e., in A and BT, ANCOVA, *p* < 0.001). No significant effect of the topological position was found on the rate of development of primary and secondary axes in any of the six species (ANCOVA, *p* > 0.20).

### Coordination of organ growth within a phytomer

**Figure 5:**
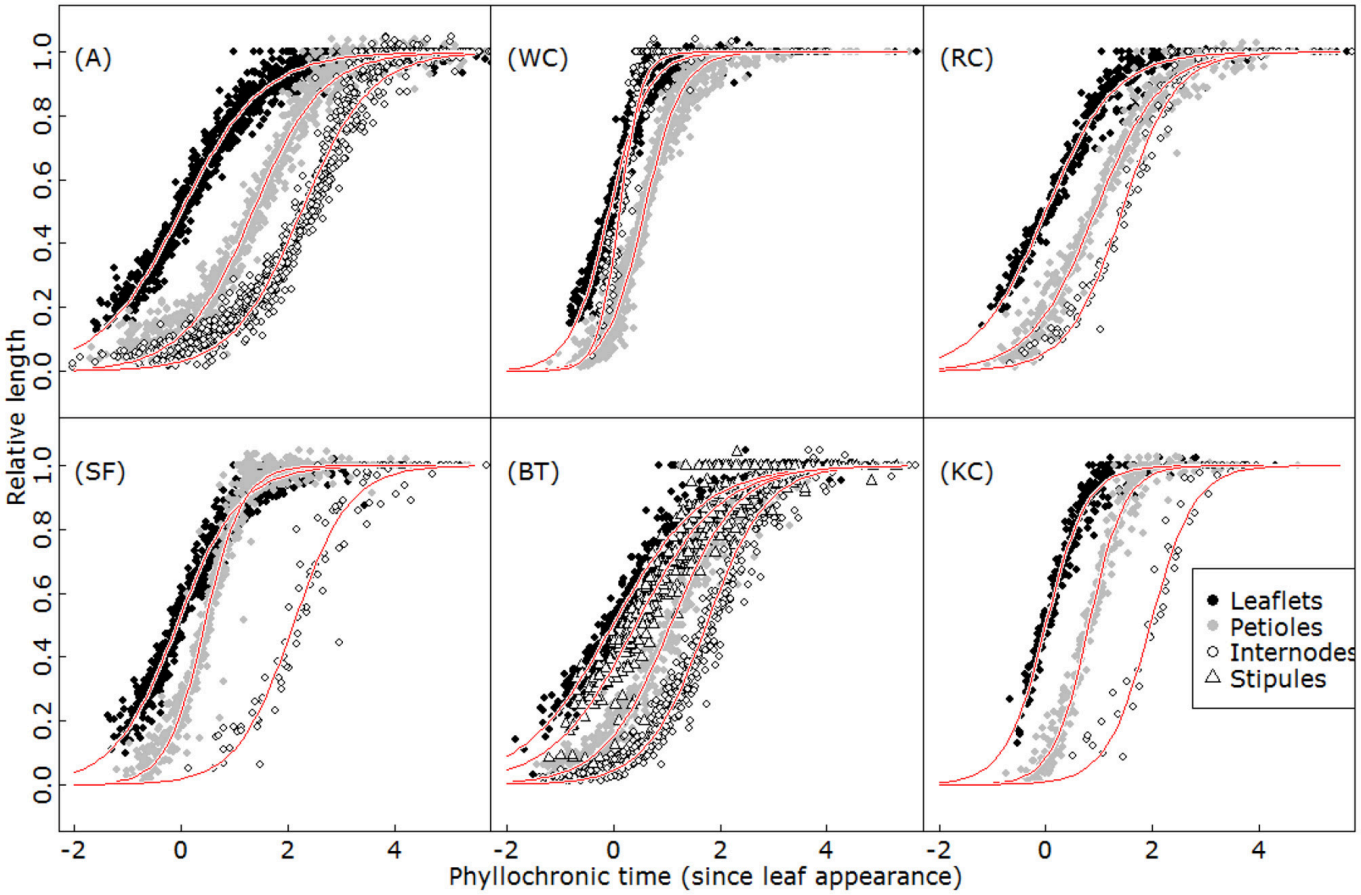
**Kinetics of relative growth for leaflets (black filled circles), petioles (gray filled circles), internodes (open circles) and stipules (open triangles) in alfalfa (A), white clover (WC), red clover (RC), sainfoin (SF), birdsfoot trefoil (BT), and kura clover (KC)**. Red lines represent the fits of a logistic function (Equation 3) for each organ type.

Once initiated, the different organs within a phytomer displayed highly conserved kinetics of expansion when expressed according to axis development (Figure 5). A strict scheduling organized the sequence of the onset of organ growth and the subsequent relative expansion of the different organs. For all of them, the time sequence of organ elongation as a function of phyllochronic time was approximated correctly using a sigmoid function (Equation 3). No significant effects of phytomer rank or order were found with respect to growth delay and maximum relative elongation rate in any of the species (*d* and *s* parameters, ANOVA, *p* > 0.51), suggesting a coordination of organ growth was conserved irrespective of the phytomer within each species.

On the other hand, the different species displayed very dissimilar organ growth coordination patterns. The differences were particularly marked concerning the maximum elongation rates and the duration of phytomer expansion (ANOVA, *p* < 0.001). Some species, such as A and BT, typically presented a slow expansion of organs relative to axis development, and phytomer growth lasting for five phyllochrons. By contrast, white clover displayed rapid expansion and a total duration limited to 2.5 phyllochrons. The species also differed in the timing and relative order of organ growth. In most cases, leaf elements elongated first (leaflets > (stipules) > petiole), followed by internode elongation. However, in white clover, the order was reversed and internodes were first to complete their elongation.

### Organ dimensions at maturity

**Figure 6:**
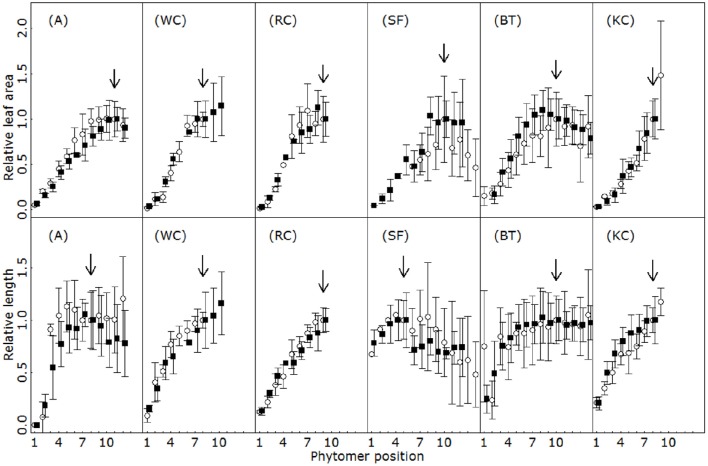
**Relative leaf area (first row) and relative length of the main spacing organ (second row, G and K: internodes; H,I,J, and L: petioles) for the first 14 phytomers on the primary axes for alfalfa (A), white clover (WC), red clover (RC), sainfoin (SF), birdsfoot trefoil (BT) and kura clover (KC)**. Vertical bars indicate standard deviation (4 to 5 replicates). Different colors indicate different experimental years. Arrows indicate the position of the rank used for the normalization of relative length and relative area. The main spacing organs were internodes in A, BF and SF and petioles in WC, RC, and KC.

For each species, the size ultimately attained by individual organs at maturity depended on the phytomer position. Figure 6 presents the changes in relative organ dimensions along the primary axes. Irrespective of species and organ type, typical vegetative shoot patterns displayed profiles that first increased in size in line with phytomer ranks, and then stabilized at a plateau value, or even decreased. Interestingly, the relative profiles remained unchanged between the experimental years and were characteristic of isolated plants in a given species. Rank by rank comparisons of relative dimensions yielded identical mean values in 50 out of 53 cases for leaves, and 57 out of 58 cases for petioles/internodes (Student test, *p* > 0.5). For each organ type, the species differed in terms of the position of the phytomer at which maximum organ size was achieved. Concerning individual leaf size for instance, up to 12 phytomers were produced before reaching the peak in SF, but only 7–8 were necessary in WC or A.

As for the actual size of organs, maximum leaf area and petiole length differed in all the species, but the ranking between species remained unaffected by the experimental year (Table 1). Significant differences were observed between years only for the maximum internode length in some of the species. This trait was slightly smaller during the second experiment in white clover and alfalfa, but not in red clover and sainfoin.

**Table 1:** **Maximum organ dimensions observed during experiments 1 and 2 for alfalfa (A), white clover (WC), red clover (RC), sainfoin (SA), birdsfoot trefoil (BT), and kura clover (KC)**.

**Organ**	**A**		**WC**		**RC**		**SF**		**BT**	**KC**	**ANOVA**					
	**2014**	**2015**	**2014**	**2015**	**2014**	**2015**	**2014**	**2015**	**2015**	**2015**	**Species**		**Year**		**Species × Year**	
											**F**	**Pr**	**F**	**Pr**	**F**	**Pr**
Petiole length	61.5 ± 4.5	72.5 ± 6.1	261 ± 31	259 ± 28	290 ± 35	255 ± 34	101 ± 34	77.5 ± 12.0	10.6 ± 0.95	208 ± 28	137.21	<0.001	1.693	0.21	1.21	0.33
Internode length	85.0 ± 3.92	56.8 ± 8.46	49.0 ± 7.53	38.1 ± 4.67	87.8 ± 4.5	84.2 ± 30.4	68.0 ± 16.3	74.1 ± 1.21	24.2 ± 8.42	62.7 ± 9.45	15.90	<0.001	6.68	<0.05	2.70	<0.05
Number of leaflets	3	3	3	3	3	3	15 ± 3.5	16.5 ± 7.9	3	3	110.50	<0.001	0.04	0.83	0.04	0.99
Leaf area	33.1 ± 8.3	28.5 ± 1.8	46 ± 5.0	46.7 ± 5.9	64.3 ± 11.0	62.2 ± 8.3	73.9 ± 32.2	58.3 ± 12.7	8.9 ± 2.2	37.7 ± 15.6	11.35	<0.001	1.23	0.28	0.53	0.66

*Average ± standard deviation (length in mm, area in cm^2^). Only the four species measured during the two experiments were included in the ANOVA*.

### Leaf allometry

Leaf size and shape varied considerably between species and phytomers. Except in sainfoin, the leaves were all trifoliolate from the second leaf on. Sainfoin presents compound leaves, with a number of leaflets that can increase up to 25 (Table 1). Despite the variability in their shapes, leaves from all the species complied with a single allometric relationship (*r*^2^ = 0.96; Supplementary Figure S2) linking leaf area (LA) with central leaflet length (L), central leaflet width (l) and the number of leaflets (n):

(5) $$\begin{array}{l} \text{LA}=0.694\times \text{L}\times \text{l}\times \text{n} \end{array}$$

### Relationships between shoot morphogenetic traits

**Figure 7:**
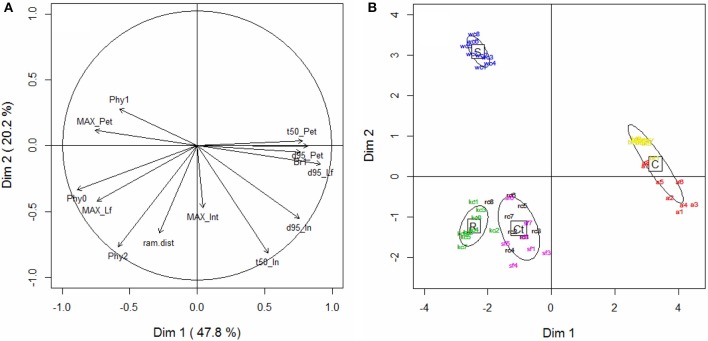
**Principal component analysis. (A)** Variables (morphogenetic traits) located on the plane defined by components 1 (horizontally) and 2 (vertically), and **(B)** individual scatterplot for component 1 and 2. See Table 2 for the names of the traits. Different colors represent individuals from different species: alfalfa (a, red), white clover (wc, blue), red clover (rc, black), sainfoin (sf, purple), birdsfoot trefoil (bt, yellow), and kura clover (kc, green). Ellipses of dispersion represent the four morphogenetic groups (stolon-formers, S; rhizome-formers, R; crown-formers tolerant to defoliation, Ct; crown-formers intolerant to defoliation, C).

The possibility of defining sets of trait values occurring concomitantly was assessed by performing PCA on the dataset defined by the major morphogenetic traits characterized during the two experiments (Table 2). The first component of this PCA (Figure 7A), which explained almost half of total variance (47.8%), was mainly determined by leaf growth traits (MAX_pet_, MAX_lf_, duration of leaf growth) and by the organogenesis of the main and primary axes (Phy0, Br1). It expressed an antagonism between the rate of phytomer production and the size and duration of expansion of leaf elements. Component 2, on the other hand, was mainly correlated to traits controlling the kinetics of internode expansion (t50_in_) and to the development of secondary branches (ram_dist, Phy2). The growth of internodes was the most able in discriminating the species in terms of their growth coordination calendar. Petiole and leaflet expansions were tightly related in all the species, but internodes could expand either before or after leaf elements. In the plane containing component 1 and component 3 (not shown), it was seen that the third component was mainly correlated to the maximum length of internodes (MAX_in_). Phy2 was also positively correlated with this third component, showing that species with the longest nodes also displayed a more vigorous development of secondary branches.

**Table 2:** **Variables used in the Principal Component Analysis presented in Figure 7**.

**Abbreviation**	**Variable**	**Unit**
Phy0	Phyllochron of the main axis	°Cd. phytomer^−1^
Br1	Primary axis production rate	Phyllochron.axis^−1^
Phy1	Phyllochron of primary axes relative to the main axis	Phytomer.phytomer^−1^
Ram.dist	Delay between phytomer production and axillary budburst	Phyllochron
d95_in	Internode expansion duration	Phyllochron
d95_pet	Petiole expansion duration	Phyllochron
d95_lf	Leaflet expansion duration	Phyllochron
t50_pet	Delay between leaflet and petiole expansion	Phyllochron
t50_in	Delay between leaflet and internode expansion	Phyllochron
MAX_pet_	Maximal petiole length at maturity	cm
MAX_in_	Maximal internode length at maturity	cm
MAX_lf_	Maximal leaf area at maturity	cm^2^

## Discussion

### Potential shoot morphogenesis followed a set of deterministic rules common to the six legume species

The morphogenesis of shoots can lead to highly differentiated plant architectures in perennial herbaceous legumes (Forde et al., 1989; Thomas, 2003). However, as species differ in their branching complexity and in the size, position and shape of their shoot organs, our results highlighted the fact that they also share a number of determinants regarding the organogenesis and growth of phytomers, the building blocks of plant architecture. The striking differences between the species we studied emerged within the framework of a common and quite delimited pattern of vegetative growth and development. This framework involved: (i) a deterministic branching pattern and a regular development of shoot axes, (ii) a coordination of organ growth at the phytomer level, and (iii) a conserved allometry of leaf shapes.

Concerning shoot organogenesis, the existence of a generalized pattern of development was supported by the fact that all the species complied with the proposed classification of shoot axes in three categories. The main axis displayed developmental characteristics distinct from primary axes (that subsequently emerged close to the plant collar) and secondary branches (emerging from axillary buds out of the collar zone), each type presenting identical phyllochrons during the different experiments and for axes at different topological positions. In two of the species (namely A and BT), the main axis and primary axes presented similar characteristics, making the classification even potentially simpler in legumes where the main axis can elongate. Given the important differences existing among dicots in terms of branching behavior and apical dominance (McSteen and Leyser, 2005), such regularity in the organogenetic process was not necessarily to be expected. Sylleptic shoot branching often occurs as a seemingly stochastic process, under the dependence of internal and environmental regulatory signals (Génard et al., 1994; Seleznyova et al., 2002; Rameau et al., 2015). Complex trophic and hormonal interplays can result in branches of the same order expressing very different characteristics depending on their position in the branching system (Lebon et al., 2004; Moreau et al., 2007), or presenting properties that change over time (e.g., increasing phyllochron, Barillot et al., 2012). Overall, the constant phyllochrons and branching delays we reported in the species we studied appeared to constitute a very simple way to characterize potential organogenesis. These characteristics may hold true mainly because we focused our attention on the period of vegetative development, during which little competition from other plant parts occurs. However, these observations were consistent with previous reports regarding herbaceous legumes (e.g., in alfalfa, Baldissera et al., 2014), and will make the characterization of new species and genotypes easier in this group of species.

The temporal coordination of organ growth also appeared to be highly conserved, irrespective of phytomer position, in all the species. Such stable calendars of expansion had previously been reported for leaves and internodes on the primary axis of different dicotyledonous species (Granier et al., 2002; Demotes-Mainard et al., 2013). Interestingly, our results suggest that this could be extended to axes of different types once time is normalized by the rate of leaf appearance on each axis (i.e., phyllochronic time). Such an approach is new for dicots but had been used successfully to account for the flexible growth pattern of grass leaves, constantly adapting to the timing of leaf appearance (Fournier et al., 2005; Zhu et al., 2014). It could probably be applied to account for the relationship between the rate of phytomer production and the timing and duration of organ growth within a phytomer on a broad range of species.

The allometric relationship found between leaf area and the product of leaf length and leaf width is currently applied in numerous species (Schwarz and Kläring, 2001; Antunes et al., 2008; Baldissera et al., 2014). Accounting for the number of leaflets was sufficient to encompass the different species within the same relationship in our study (Equation 5). This indicates that a common shape coefficient could be applied to expanded leaflets from the different legume species (Prévot et al., 1991). However, considering both leaf length and leaf width was a necessity to establish this common relationship, because some dissociation exists between the longitudinal and lateral expansion of leaf laminas (e.g., the former being mediated by brassinosteroids without any effect on the latter; Nakaya et al., 2002), resulting in length to width ratios that vary as a function of phytomer position and species.

On the other hand, no clear pattern governing organ dimension at maturity, and which could encompass all the species, emerged at the axis level. Typical leaf and internode length profiles reached a maximum value at an intermediate position along the axis, a situation that is relatively ubiquitous in vascular plants (Allsopp, 1967; Villani and Demason, 2000). These positions were similar between experiments in a given species, but they changed dramatically depending on organ type and between species. Furthermore, the maximum dimension attained by internodes changed between two of the experiments. As water and nutrients were supplied without restriction, these differences could have resulted from the slightly different light regimes prevailing in the different years, or could have been caused by greater evaporative demand affecting organ expansion in 2015 (Supplementary Table S1, Tardieu et al., 2005).

### Differences in trait values determined the contrasting morphogenetic strategies

Despite this common developmental pattern, the species sampled did produce contrasting shoot architectures. Even without considering geometric features (such as leaf angles, shoot bearing, etc.), the values for component traits of shoot morphogenesis and which accounted for phytomer production (phyllochrons, delay of branching) and organ growth (delays of expansion, relative expansion rate, maximum organ dimension) explained the emergence of four morphogenetic groups (Figure 7B) that partially matched those identified by Thomas (2003). Only kura clover, which is in fact a rhizome-forming species, was closely related with two crown-forming species producing a short main axis (RC and SF). As previously shown (Genrich et al., 1998; Black et al., 2006), kura clover was very slow to develop and did not express its rhizomatous growth habit during the period studied. The primary axes characterized from rhizome cuttings in the third experiments did not differ from those emerging from collar in the second experiment. The developmental pattern of KC shoots thus appeared very similar to red clover in our conditions, explaining the close classification of the two species.

The main morphogenetic traits associated with this classification were distinguished along two PCA axes. A first dimension represented the strategies of leaf area production, reliant on either the production of numerous small leaves or the expansion of a fewer large leaves. Such a trade-off between growth and development is common during morphogenesis and has, for instance, been reported in grass tillers (leaf length vs. phyllochron and tillering rate; Gautier et al., 1999; Nelson, 2000), and in roots (root elongation rate vs. branching ability: Pagès, 2014). In forage legumes, this could be a key element in controlling the rate of regrowth in a mixture after defoliation (e.g., in white clover and alfalfa: Davies, 2001; Annicchiarico, 2003; Annicchiarico et al., 2015). A second dimension mainly distinguished species in terms of their growth coordination patterns, promoting either early internode growth or the early growth of leaf elements (including petioles) as a primary option to colonize space and expand vegetative structures. All species but white clover favored the growth of leaf elements, which is consistent with a pre-emptive strategy for light acquisition that could be expected in crown-formers with limited prospecting ability. Under such local competition, rapid plant leaf area development indeed appears to be the most successful trait to capture light (Louarn et al., 2012). On the other hand, white clover develops primary axes by first sensing its environment through internode elongation. This particular trait could be an adaptation to achieve horizontal prospection for light gaps, as white clover tends to be a light foraging species rather than a competitive one (de Kroons and Hutchings, 1995). Similar growth coordination patterns are observed in lianas (e.g., grapevine, Louarn, 2005) and in invasive species sprouting through long cane emission (e.g., rubus sp., Amor, 1974), thus making such an hypothesis plausible.

A degree of redundancy between several of the morphogenetic traits was apparent (e.g., high correlations between petiole and leaflet size, or between the size of these organs and their duration of expansion), which suggests opportunities to simplify the characterization of morphogenetic strategies. Overall, however, the combined values of these traits enabled the discrimination and classification of the species as a function of their known ecological behaviors.

### Interests and limitations of such a framework for plant phenotyping and modeling

Identifying a robust framework that enables the description, analysis and prediction of plant morphogenesis is central to developing efficient phenotyping approaches for breeding (Granier et al., 2002; Moreau et al., 2006) and the diagnosis of plant stress (e.g., Pellegrino et al., 2005). The framework for potential morphogenesis we have discussed above represents a first step toward achieving both of these goals with respect to perennial herbaceous legumes, many of which have been little characterized to date. Setting up such a potential framework in absence of competition is particularly important to identify ontogenic patterns of development and to disentangle the contradictory effects of neighbors on plant morphology and resource acquisition (Lemaire and Millard, 1999).

Clearly, our approach is currently limited to the vegetative period of development, and the interactions which may take place during flowering or the development of reproductive organs at later stages are not taken into account. In most forage species, regular harvests of shoot biomass over a season involve several cuts of primary shoot axes, and a succession of vegetative development from crown and the remaining lateral buds, thus making particularly relevant the vegetative framework presented. However, in prostrate species such as white clover, or in those with a terminal flowering (Thomas, 2003), interactions with the reproductive cycle will occur at some point, and integrating a reproductive dimension in the framework would most likely improve its robustness (e.g., Moreau et al., 2007). Similarly, the development of nodal roots has been shown to interact with shoot organogenesis in aging clover plants (Thomas et al., 2014), and a whole plant appraisal of morphogenesis, considering both shoot and root morphogenetic traits (Pagès, 2014) would deserve exploration. In any case, the set of deterministic rules identified in this study could serve as an initial benchmark for the future development of a more comprehensive framework. As a potential morphogenetic model, it could also serve as a baseline to analyse the responses of vegetative development to environmental stresses (Belaygue et al., 1996; Lebon et al., 2006; Baldissera et al., 2014), and to compare on a quantitative basis a broad range of species and genotypes with respect to their morphogenetic strategies (Louarn et al., 2007).

At present, the framework provides a generic approach to break down the component traits of competitive ability aboveground (i.e., the components of leaf area expansion and height acquisition: Barillot et al., 2011; Louarn et al., 2012). This is of particular value because: (i) the genetic component of competitive ability has been shown to be more tightly related to component traits of morphogenesis than to integrated traits (Annicchiarico et al., 1999), (ii) such a framework could help to highlight specific combinations of traits associated with a particular morphogenetic group, even in species for which the amount of background studies is limited, and (iii) the knowledge of component traits associated with competitive ability is expected to increase the efficiency of pure stand selection that targets mixed stand conditions, thus offering opportunities to reduce costs during the selection process for multi-species grasslands (Annicchiarico, 2003). Finally, such a quantitative approach could also readily be integrated as a component in mixed grassland models (Soussana et al., 2012), so as to enable better consideration of the diversity of forms that legume components could accommodate, and to improve the prediction of their persistence in mixtures.

## Author contributions

LF and GL designed the experiments, conducted measurements and performed data analyses. IL and GL rose funding for these research. All of the authors contributed to writing the manuscript.

### Conflict of interest statement

The authors declare that the research was conducted in the absence of any commercial or financial relationships that could be construed as a potential conflict of interest.
