# Supplementary material for: A Conserved Potential Development Framework Applies to Shoots of Legume Species with Contrasting Morphogenetic Strategies
Source: Front Plant Sci. 2017 Mar 27;8:405. doi: 10.3389/fpls.2017.00405 (PMC5366346; doi:10.3389/fpls.2017.00405)
Supplement: Supplementary file 1 [file Table1.doc]

**SUPPLEMENTARY MATERIAL**

Sup. Table S1: Summary of the environmental conditions prevailing during the three experiments.

| month.year | Air temperature (°C) | | | | PPFD (mol.m⁻².d⁻¹) | | | | VPD (kPa) | | | |
| --- | --- | --- | --- | --- | --- | --- | --- | --- | --- | --- | --- | --- |
|  | mean ± sd | | min | max | mean ± sd | | min | max | mean ± sd | | min | max |
| Feb. 2014 | 18.1 | ±0.6 | 17.4 | 19.1 | 8.2 | ±2 | 5.3 | 11.6 | 0.82 | ±0.1 | 0.6 | 1 |
| Mar. 2014 | 19.7 | ±1.3 | 16.6 | 21.8 | 13.2 | ±4.9 | 6.1 | 21.7 | 1.0 | ±0.2 | 0.7 | 1.5 |
| Apr. 2014 | 21.6 | ±1.3 | 19.0 | 23.8 | 17.1 | ±6.6 | 2.4 | 26.8 | 1.1 | ±0.2 | 0.6 | 1.6 |
| Feb. 2015 | 16.7 | ±0.8 | 15.2 | 18.1 | 7.1 | ±1.8 | 10.6 | 11.3 | 0.8 | ±0.1 | 0.7 | 1.1 |
| Mar. 2015 | 18.3 | ±1.5 | 15.6 | 21.7 | 11.1 | ±3.8 | 10.3 | 16.3 | 1.0 | ±0.2 | 0.6 | 1.5 |
| Apr. 2015 | 21.0 | ±2.2 | 17.4 | 25.3 | 17.4 | ±6.5 | 5.9 | 25.3 | 1.3 | ±0.4 | 0.6 | 2 |
| Nov. 2016 | 18.1 | ±1.7 | 13.4 | 20.4 | 5.8 | ±2.2 | 1.0 | 9.8 | 0.5 | ±0.2 | 0.2 | 0.7 |
| Dec. 2016 | 17.6 | ±1.5 | 14.9 | 19.9 | 4.1 | ±1.7 | 1.0 | 6.1 | 0.5 | ±0.1 | 0.4 | 0.7 |
| Jan. 2017 | 16.1 | ±1.8 | 12.8 | 20.0 | 4.3 | ±2 | 0.6 | 7.5 | 0.5 | ±0.1 | 0.4 | 0.6 |
| Fev. 2017 | 18.4 | ±1.3 | 16.0 | 20.5 | 6.5 | ±2.7 | 1.6 | 11.1 | 0.6 | ±0.2 | 0.4 | 0.7 |

Sup. Table S2: Parameters of the beta function (Eq. 1) used for the temperature response in alfalfa (A), white clover (WC), red clover (RC), sainfoin (SF), birdsfoot trefoil (BT), and kura clover (KC). Data from the literature were aggregated according to the method proposed by Parent and Tardieu (2012). Measurements of seedling growth were selected and normalized at a reference temperature equal to 20°C for each dataset. The *nls* procedure from the R software was used to identify parameter values.

| Species | Tmax | Tmin | q | References |
| --- | --- | --- | --- | --- |
| A | 39.4 | -7.6 | 3.22 | [1, 8, 12, 13] ; Faverjon (unpublished data) |
| WC | 38.7 | -1.79 | 2.17 | [3, 5, 7, 9, 12] ; Faverjon (unpublished data) |
| RC | 37.1 | -33.3 | 7.55 | [11] ; Faverjon (unpublished data) |
| BT | 42.8 | 1.96 | 1.59 | [6, 8, 11] ; Faverjon (unpublished data) |
| SF | 39.4 | -7.6 | 3.22 | [1, 2] |
| KC | 37.1 | 5 | 1.24 | [4, 5] ; Faverjon (unpublished data) |

**List of references**:

[1] Ahmed,L., 2015. Analysis of Inter- and Intra- Specific Variability of Five Pasture Species in Response to Temperature During Germination and Initial Heterotrophic Growth. PhD Thesis, University of Poitiers, France.

[2] Allahmoradi, Pezhman, Mokhtar Ghobadi, and Shayesteh Taherabadi. "Assessing Cardinal Temperature for Germination in Coriander (Coriandrum sativum), Sainfoin (Onobrychis vicifolia) and Bitter Vetch (Vicia ervilia)." Annual Review & Research in Biology 3.4 (2013): 881-887.

[3] Beinhart, G. 1963. "Effects of environment on meristematic development, leaf area, and growth of white clover." Crop science 3.3 (1963): 209-213.

[4] Black, A., D. Moot, and R. Lucas. 2002. “Seedling Development and Growth of White Clover, Caucasian Clover and Perennial Ryegrass Grown in Field and Controlled Environments.” *Proceedings of the New Zealand Grassland Association*. http://researcharchive.lincoln.ac.nz/handle/10182/4505.

[5] Black, A. D., Moot, D. J., & Lucas, R. J. 2006. Development and growth characteristics of Caucasian and white clover seedlings, compared with perennial ryegrass. Grass and Forage Science, 61(4), 442-453.

[6] Hur, S. N., and C. J. Nelson. 1985. "Temperature effects on germination of birdsfoot trefoil and seombadi." Agronomy Journal 77(4): 557-560.

[7] Lonati, Michele, et al. "Thermal time requirements for germination, emergence and seedling development of adventive legume and grass species." New Zealand Journal of Agricultural Research 52.1 (2009): 17-29.

[8] Masiunas, J. B., & Carpenter, P. L. 1984. Radicle growth of grasses and legumes in response to temperature. HortScience.

[9] Moot, D. J., Scott, W. R., Roy, A. M., & Nicholls, A. C. 2000. Base temperature and thermal time requirements for germination and emergence of temperate pasture species. New Zealand Journal of Agricultural Research, 43(1), 15-25.

[10] Parent B, Tardieu F. 2012. Temperature responses of developmental processes have not been affected by breeding in different ecological areas for 17 crop species. New Phytologist 194, 760‑74.

[11] Qualls, M., & Cooper, C. S. 1968. Germination, Growth, and Respieration Rates of Birdsfoot Trefoil at Three Temperatures During the Early Non-Photosynthetic Stage of Development. Crop Science, 8(6), 758-760.

[12] Sakanoue, S. 2010. Thermal time approach to predicting seedling emergence dates of red clover, white clover and lucerne in farm fields. Grass and Forage Science 65.2 (2010): 212-219.

[13] Zaka, S., Ahmed, L. Q., Escobar-Gutiérrez, A. J., Gastal, F., Julier, B., & Louarn, G. 2017. How variable are non-linear developmental responses to temperature in two perennial forage species?. Agricultural and Forest Meteorology, 232, 433-442.


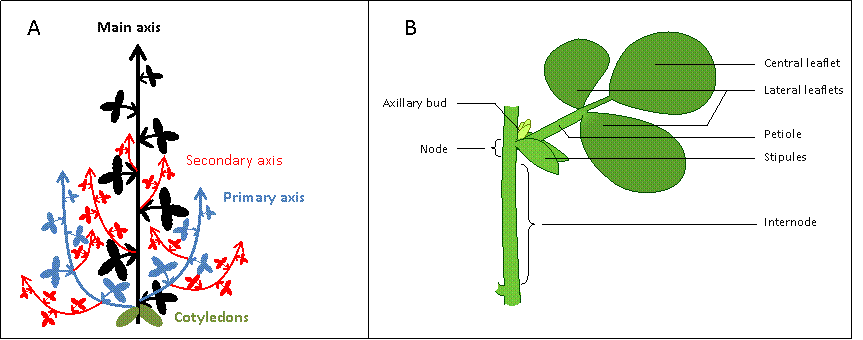


Sup. Figure S1: Generalized classification of shoot axes and terminology used to describe forage legume shoots (A) and the organization of organs within a phytomer (B).


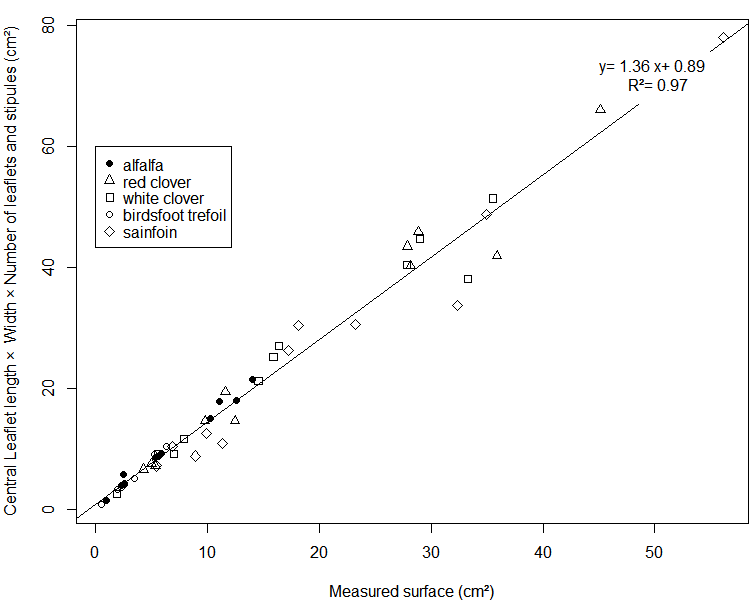


Sup. Figure S2: Relationships between the surface area of individual leaves and the product of the length, and width of the central leaflet by the number of leaflets (Eq. 4) for the six legume species studied.
